# Association between sleep quality and benign prostate hyperplasia among middle-aged and older men in India

**DOI:** 10.1186/s12889-023-15972-6

**Published:** 2023-06-14

**Authors:** Kai Ma, Qiang Dong

**Affiliations:** Department of Urology, institution of Urology, West China Hospital of Sichuan University, Chengdu, 610000 Sichuan Province China

## Abstract

**Background:**

The association between sleep quality and benign prostate hyperplasia (BPH) has rarely been studied. The aim of this study was to examine the relationship between sleep quality and BPH among middle-aged and older men in India.

**Methods:**

This study used data from men over 45 years old in Wave 1 (2017–2018) of the Longitudinal Aging Study in India (LASI). Benign prostate hyperplasia was self-reported, and sleep symptoms were assessed using five questions modified from the Jenkins Sleep Scale. A total of 30,909 male participants were finally included. Multivariate logistic regression analysis, subgroup analysis, and interaction tests were performed.

**Results:**

Total 453 (1.49%) men reported benign prostatic hyperplasia and have higher sleep quality score (9.25 ± 3.89 vs. 8.13 ± 3.46). The results revealed that the sleep quality score and risk of benign prostatic hyperplasia were significantly correlated after adjusting for all confounding factors (OR:1.057, 95% CI: 1.031–1.084, p < 0.001]. After dividing people into four groups based on the quartile of sleep quality scores, compared with the first quartile group, the third quartile group was 1.32 times, and the fourth quartile group was 1.615 times more likely to develop benign prostate hyperplasia. A significant interaction effect of alcohol consumption was observed. (p for interaction < 0.05).

**Conclusion:**

Worse sleep quality was significantly associated with a higher incidence of benign prostatic hyperplasia among middle-aged and older Indian men. A further prospective study is needed to clarify this association and explore potential mechanisms.

**Supplementary Information:**

The online version contains supplementary material available at 10.1186/s12889-023-15972-6.

## Introduction

Benign Prostatic Hyperplasia (BPH) is a disease that usually occurs in older men and causes urination dysfunction. Traditionally, it happens after the age of 40 and increases with age. At the age of 80, its prevalence rate is as high as 83%. [1, 2] Due to the growth of stromal cells and prostate epithelial cells, the urethra is physically compressed by the prostate, resulting in Bladder Outlet Obstruction (BOO). Moreover, this caused Lower Urinary Tract Symptoms (LUTS), which include symptoms of obstructive voiding (hesitancy, straining, weak stream, sensation of incomplete emptying) or irritable voiding (frequency, urgency, urge incontinence). [3, 4]

Numerous risk factors have been identified to increase the incidence of benign prostate hyperplasia,, including obesity, metabolic syndrome, inflammation, sex hormone levels, and so on. [4] At the same time, sleep promotes the growth of the central nervous system and the restoration of physical functioning. [5] Limited sleep has been linked to immune system abnormalities, metabolism, and hormones in humans and animals. [6–8] Therefore, sleep disorders might play a role in influencing the prostate condition through alterations in circadian regulation, steroid hormone action, nervous system input, and inflammation. [9, 10] According to earlier studies, poor sleep quality has been linked to prostate disease. The correlation between sleep disorders and BPH was reported by Li et al. [11] and Yang et al. [12] among Chinese men. A cohort study conducted by Branch et al. [13] also showed that worse sleep quality was suggestively associated with the development of lower urinary tract symptoms regardless of men with or without LUTS at baseline.

However, the association between BPH risk and sleep quality in Indian men is still unclear. Therefore, our study aimed to examine any connections between sleep quality and the occurrence of BPH. The high frequency of BPH significantly exacerbates the suffering of these individuals as well as the health and financial burden on our society. [14] The findings may contribute to developing new methods for preventing BPH and raising the standard of living for the aging population.

## Method

### Data and participants

**Fig. 1:**
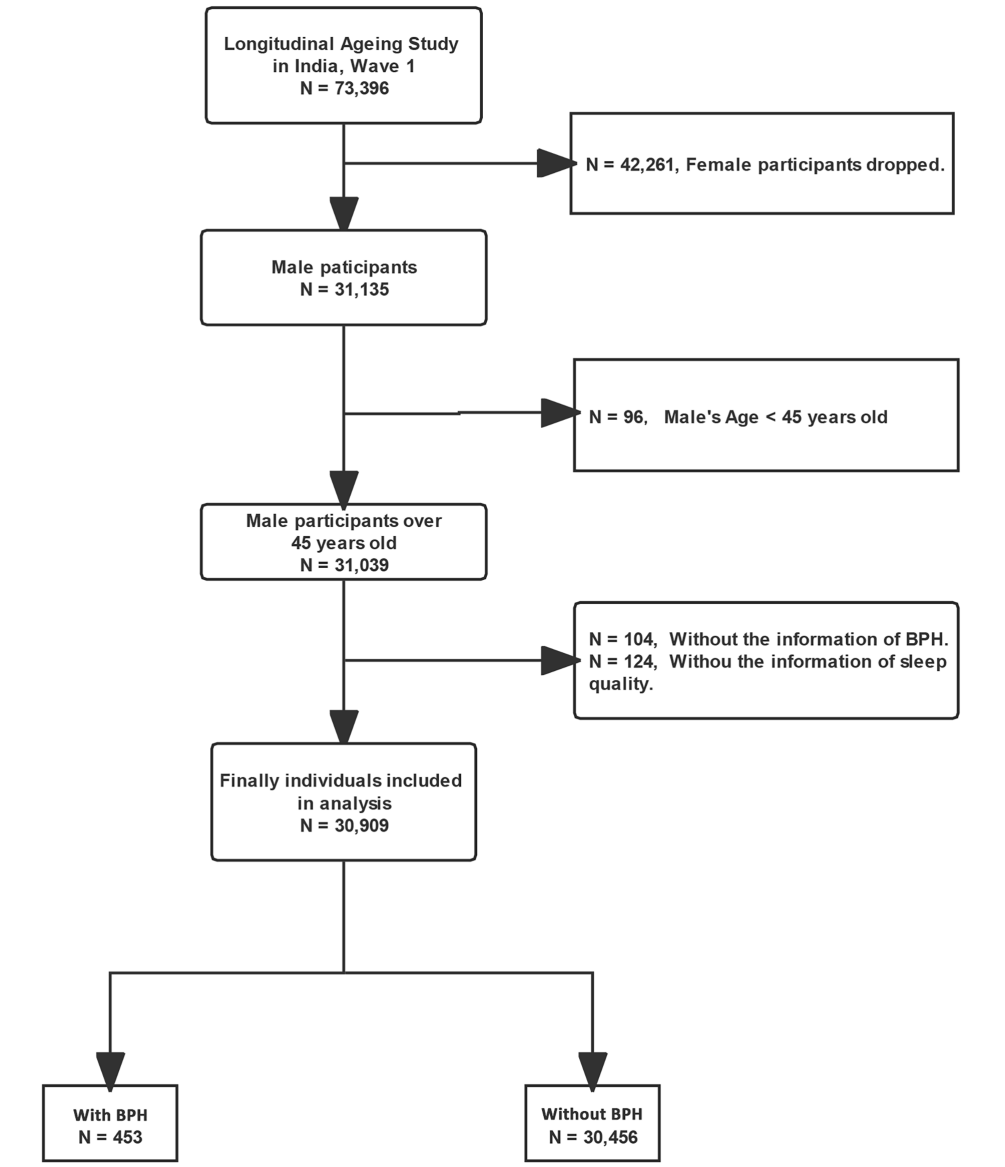
Flowchart of sample selection. BPH: benign prostatic hyperplasia

The current study’s data were derived from the Longitudinal Aging Study in India (LASI), Wave I, 2017-18. This wave enrolled 73,396 participants over 45 years old and their spouses regardless of age in the whole country. Details on survey tools, data collection techniques, and sampling methodology can be found in a previous study. [15] This study focused on the association between sleep quality and benign prostatic hyperplasia among middle-aged and older men; only men over 45 years older were included in the analysis. Females, men under 45, missing values for BPH and sleep quality were excluded. Figure 1 shows the process flow of this study’s samples.

### Definition of sleep quality and outcome

Five questions modified from the Jenkins Sleep Scale were used to evaluate sleep issues in the LASI survey. [16] (i)“How often do you have trouble falling asleep?” (ii) “How often did you have trouble getting back to sleep after waking up during the night?” (iii) “How often do you have trouble with waking up too early and not being able to fall asleep again?” (iv) “How often did you feel unrested during the day regardless of the number of hours of sleep you had?” (v) “How often did you take a nap during the day?” The code 1,2,3,4 stood for “never, rarely (1–2 nights per week), occasionally (3–4 nights per week), and frequently (5 or more nights per week)”. The Cronbach’s alpha of this scale was 0.83, which means good reliability. We used a total score of five questions to represent sleep quality, which ranges from 5 to 20. A lower number was considered better sleep quality, similar to the preliminary study. [17]

Benign prostate hyperplasia was self-reported and assessed by asking, “Have you ever been diagnosed with any of the following urogenital conditions or diseases with options including BPH?”

### Covariates

Some variables that have been demonstrated to influence benign prostate hyperplasia were added as covariates. Age was coded in years. Education was divided into never educated, middle school or under, secondary and.

higher secondary, and above higher secondary. Considering India’s unique racial system, we divided it into scheduled caste, scheduled tribe, other backward class, and no or other castes. Marital status was divided into married or partnered, widowed, and others. Rural and urban areas classified the place of residence. BMI was divided into underweight (< 18.5 kg/m^2^), normal (18.5–25 kg/m^2^), and overweight (≥ 25 kg/m^2^). Tobacco consumption (never, light, moderate, vigorous), alcohol consumption (never, light, moderate, heavy), and physical activity (never, seldom, sometimes, frequent) were categorized based on their frequency. In addition, we extracted several chronic diseases as potential confounders defined by the question “Has any health professional ever diagnosed you with the following chronic conditions or diseases?”, which contains history of hypertension, high cholesterol, cancer, chronic heart disease, pulmonary disease, stroke, bone or joint diseases, and any neurological problem. The missing values of covariates were interpolated using multivariate imputation based on predictive mean matching methods.

### Statistical analysis

Continuous variables are represented by means and standard deviations in the baseline characteristics, while rates and percentages represent categorical variables. For continuous variables, p-values were obtained using the Kruskal–Wallis rank-sum test, which did not need the data to satisfy normality and homogeneity of variance with greater applicability, and chi-square tests for categorical variables. If the theoretical number was < 10, Fisher’s exact test was used.

First, we regarded the sleep quality score as a continuous variable. Then, since the sleep quality score was left-skewed distribution, we divided participants into four groups by quartile of their sleep quality score: the individuals in first quartile of sleep quality score (Q1 group, sleep quality score = 5 ); the individuals in second quartile of sleep quality score (Q2 group, 5 < sleep quality score ≤ 7 ); the individuals in third quality of sleep quality score (Q3 group, 7 < sleep quality score ≤ 10 ); the individuals in the fourth quality of sleep quality score (Q4 group, 10 < sleep quality score ≤ 20 ). We used multivariate logistic regression analyses to explore the association between benign prostate hyperplasia and sleep quality scores, adjusting for different covariates to reduce the influence of other confounding factors on the study results. The first model was only adjusted for age (Model I); the second model was adjusted for age, education level, marital status, place of residence, and caste (Model II); the third model was adjusted for age, education level, marital status, place of residence, caste, alcohol consumption, tobacco consumption, and physical activity (Model III); and the fully adjusted models were adjusted for age, education level, marital status, place of residence, caste, alcohol consumption, tobacco consumption, BMI, physical activity, comorbidities (diabetes, hypertension, high cholesterol, cancer, chronic heart disease, chronic pulmonary disease, stroke, bone or joint diseases, any neurological or psychiatric problem.)(Model IV).

To assess the heterogeneity of the relationship between sleep quality and benign prostatic hyperplasia stratified by covariates (such as age, BMI, alcohol intake, and tobacco consumption), we ran interaction analyses. Middle age was defined as between the ages of 45 and 60, and older age was defined as over 60 years old. Stratified logistic regression models were used for the subgroup analyses, and the p for interaction was obtained using the log-likelihood ratio test to examine the differences between models with and without the interaction of covariates. All statistical results in this study were completed by R software, and a p-value < 0.05 was considered to be statistically significant.

## Result

### Study population

This study finally included a total of 30,909 male participants. Table 1 lists the individuals’ baseline characteristics. At the baseline level, males with benign prostatic hyperplasia seem to have higher sleep quality scores than men without BPH (sleep quality score: 8.13 ± 3.46 vs. 9.25 ± 3.89), and the people divided into third quartile and fourth quartile of sleep quality groups was more in BPH groups. (Q3: 25.39% vs. 22.89%; Q4: 34.44% vs. 22.76%). At the same time the average age of BPH group was older than non-BPH group. (60.04 ± 10.65 vs. 64.32 ± 11.29). In addition, there were significant differences in caste/tribe, educational levels, alcohol consumption, hypertension, diabetes, high cholesterol, chronic heart disease, and bone or joint disease between the group with BPH and the group without this disorder. (P < 0.001).

**Table 1:** Baseline characteristics for participants

		BPH		p
		No	Yes	
		(n = 30,456)	(n = 453)	
age, year (mean ± SD)		60.04 ± 10.65	64.32 ± 11.29	< 0.001
sleep quality (mean ± SD)		8.13 ± 3.46	9.25 ± 3.89	< 0.001
sleep quality quartiles(%)				
	Q1	10,149 (33.32)	109 (24.06)	< 0.001
	Q2	6406 (21.03)	73 (16.11)	
	Q3	6970 (22.89)	115 (25.39)	
	Q4	6931 (22.76)	156 (34.44)	
caste tribe				
	Scheduled caste	4987 (16.50)	70 (15.52)	< 0.001
	Scheduled trible	5400 (17.86)	38 (8.43)	
	Other backward class	11,627 (38.47)	160 (35.48)	
	No or other caste	8213 (27.17)	183 (40.58)	
residence (%)				
	Rural	19,983 (65.61)	287 (63.36)	0.34
	Urban	10,473 (34.39)	166 (36.64)	
marital status (%)				
	Married	26,654 (87.52)	392 (86.53)	0.219
	Widowed	2775 (9.11)	50 (11.04)	
	Others	1026 (3.37)	11 (2.43)	
education level (%)				
	Never	9551 (31.36)	119 (26.27)	< 0.001
	Middle school or under	12,662 (41.57)	164 (36.20)	
	Secondary and higher secondary	5904 (19.39)	123 (27.15)	
	Above higher secondary	2339 (7.68)	47 (10.38)	
BMI (%)				
	underweight	5230 (19.11)	61 (14.70)	0.076
	normal	15,563 (56.86)	249 (60.00)	
	overweight	6580 (24.04)	105 (25.30)	
tobacco consumption (%)				
	Never	13,521 (44.69)	194 (43.21)	0.29
	light	8317 (27.49)	113 (25.17)	
	moderate	6450 (21.32)	106 (23.61)	
	vigorous	1965 (6.50)	36 (8.02)	
alcohol consumption (%)				
	Never	20,042 (66.24)	286 (63.84)	< 0.001
	light	5849 (19.33)	118 (26.34)	
	moderate	2918 (9.64)	29 (6.47)	
	heavy	1447 (4.78)	15 (3.35)	
physical activity (%)				
	never	15,374 (50.81)	244 (54.34)	0.049
	seldom	2886 (9.54)	50 (11.14)	
	sometimes	2555 (8.44)	42 (9.35)	
	frequent	9440 (31.20)	113 (25.17)	
Hypertension (%)				
	no	22,887 (75.17)	285 (62.91)	< 0.001
	yes	7562 (24.83)	168 (37.09)	
Diabetes (%)				
	no	26,423 (86.79)	360 (79.47)	< 0.001
	yes	4022 (13.21)	93 (20.53)	
High cholesterol (%)				
	no	29,513 (96.91)	421 (92.94)	< 0.001
	yes	940 (3.09)	32 (7.06)	
Tumor (%)				
	no	30,295 (99.48)	447 (98.68)	0.042
	yes	157 (0.52)	6 (1.32)	
Chronic lung disease (%)				
	no	28,597 (93.91)	411 (90.73)	0.007
	yes	1856 (6.09)	42 (9.27)	
Chronic heart diseases (%)				
	no	29,201 (95.89)	415 (91.61)	< 0.001
	yes	1252 (4.11)	38 (8.39)	
Stroke (%)				
	no	29,745 (97.68)	442 (97.57)	1
	yes	708 (2.32)	11 (2.43)	
Bone or joint diseases (%)				
	no	27,060 (88.86)	361 (79.69)	< 0.001
	yes	3393 (11.14)	92 (20.31)	
Neurological or psychiatric problem (%)				
	no	29,761 (97.74)	437 (96.47)	0.1
	yes	688 (2.26)	16 (3.53)	

BPH: benign prostatic hyperplasia, SD: standard deviation, Q1: the individuals in first quartile of sleep quality score, Q2: the individuals in second quartile of sleep quality score, Q3: the individuals in third quartile of sleep quality score, Q4: the individuals in fourth quartile of sleep quality score, BMI: Body Mass Index Mean ± SD for continuous variables: P value was calculated by Kruskal Wallis rank-sum test, Number (%) for categorical variables: P value was calculated by chi-square test

### Association between sleep quality and BPH

The results of multivariate logistic regression analyses of the correlation between sleep quality and BPH risk among participants were shown in Table 2. We found that the sleep quality score was significantly related to risk of BPH (OR: 1.084; 95% CI: 1.059–1.109, P < 0.001) in the crude model. Further adjusting confounding variables attenuated this association. However, it remained significant in Adjusted IV models with OR:1.057 (95% CI: 1.057–1.084). When we divided men into four parts based on the quartile of sleep quality score, it was found that the third quartile (Q3) group and the fourth quartile (Q4) group had a higher risk of developing benign prostatic hyperplasia than the first of quartile (Q1) group in all models (all the p values < 0.05). In the fully adjusted model (Adjusted IV model), the individuals in third quartile of sleep quality score were 1.32 times, and the individuals in fourth quartile of sleep quality score were 1.615 times more likely to develop benign prostate hyperplasia than individuals in first quartile of sleep quality score.

**Table 2:** Associations between sleep quality and Benign prostate hyperplasia

		Crude Model	P-value	Adjusted I	P-value	Adjusted II	P-value	Adjusted III	P-value	Adjusted IV	P-value
		OR (95%CI)		OR (95%CI)		OR (95%CI)		OR (95%CI)		OR (95%CI)	
Sleep Quality score		1.084	< 0.001	1.068	< 0.001	1.07	< 0.001	1.068	< 0.001	1.057	< 0.001
		(1.059, 1.109)		(1.043, 1.04)		(1.044, 1.096)		(1.043, 1.095)		(1.031, 1.084)	
Sleep Quality Quartile											
	Q1	reference		reference		reference		reference		reference	
	Q2	1.061	0.697	1.031	0.841	1.037	0.812	1.008	0.96	0.975	0.871
		(0.788, 1.429)		(0.765, 1.39)		(0.769, 1.398)		(0.747, 1.359)		(0.723, 1.317)	
	Q3	1.536	0.001	1.41	0.011	1.435	0.008	1.391	0.015	1.32	0.042
		(1.18, 2)		(1.082, 1.838)		(1.1, 1.872)		(1.066, 1.816)		(1.01, 1.725)	
	Q4	2.096	< 0.001	1.827	< 0.001	1.841	< 0.001	1.795	< 0.001	1.615	< 0.001
		(1.638, 2.682)		(1.423, 2.345)		(1.432, 2.367)		(1.395, 2.31)		(1.25, 2.087)	

Multivariate logistic regression was used to identify the association between sleep quality and BPH. Participants were divided into four groups base on the quartile of sleep quality scores. Q1: men with the first quartile of sleep quality score; Q2: men with the second quartile of sleep quality score; Q3: men with the third quartile of sleep quality score; Q4: men with the fourth quartile of sleep quality score

The Q1 was set as the reference group. Crude model: non adjusted confounding variable. Adjusted 1: adjusting for age; Adjusted II: further adjusting for education level, marital status, place of residence, caste; Adjusted III: further adjusting for alcohol consumption, tobacco consumption and physical activity based on Adjusted II; Adjusted IV: further adjusting for BMI and comorbidities (diabetes, hypertension, high cholesterol, cancer, chronic heart disease, chronic pulmonary disease, stroke, bone or joint diseases, any neurological or psychiatric problem) based on Adjusted III; OR: odd ratio; CI: confidence interval; Q: quartile; BMI: body mass index

### Subgroup analysis

**Fig. 2:**
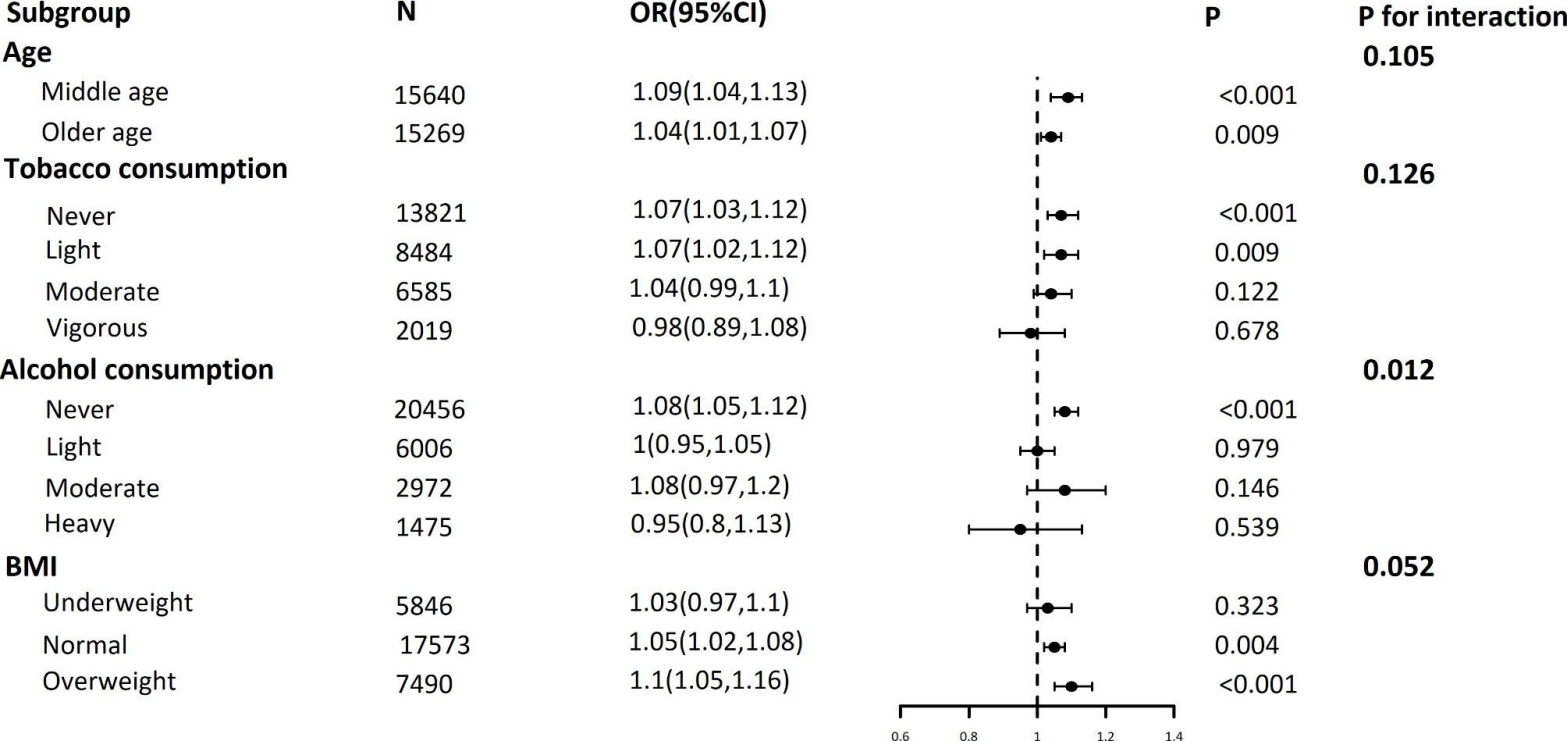
Subgroup analysis between sleep quality score and benign prostatic hyperplasia. Middle age: 45 ≤ age <60 years old; older age: age ≥ 60 years old; OR: odds ratio; 95% CI: 95% Confidence interval; BMI: body mass index; underweight: BMI < 18.5 kg/m^2^; normal: 18.5 kg/m^2^ ≤ BMI <25 kg/m^2^; overweight: BMI ≥ 25 kg/m^2^. Model was adjusted for age, education level, marital status, place of residence, caste, alcohol consumption, tobacco consumption, physical activity, BMI, comorbidities (diabetes, hypertension, high cholesterol, cancer, chronic heart disease, chronic pulmonary disease, stroke, bone or joint diseases, any neurological or psychiatric problem) except the stratified variable. P for interaction was obtained using the log-likelihood ratio test

Subgroup analysis (Fig. 2) showed that the sleep quality score was significantly associated with the risk of benign prostatic hyperplasia among the middle-aged or older individuals. Furthermore, this association was stable in the never or light consumption of tobacco, never consumption of alcohol, and normal or overweight BMI groups. (P < 0.05) The interaction test shows that alcohol consumption affects the relationship between sleep quality and BPH. (P for interaction < 0.05).

## Discussion

India’s population is aging quickly. The proportion of persons 60 and older will nearly double between 2001 and 2031, reaching an estimated 20% by the middle of the century. [18] BPH was a common disease, and its causes of lower urinary tract symptoms significantly impacted middle-aged and older men’s daily lives. Numerous studies have examined its risk factors from various angles, but only some have examined the function of sleep in the development of BPH, which is what we do in this study. Our study demonstrated that poor sleep quality was significantly associated with higher BPH prevalence among middle-aged and older adults in India.

A previous prospective cohort study by Araujo et al. [19] revealed that participants who reported poor sleep quality and sleep restriction at baseline had consistently higher risks of developing urological symptoms, including lower urinary tract symptoms and nocturia. A study conducted by Scovell et al. [20] focused on men working nonstandard shifts and.

reported that people with difficulties falling, falling back asleep, or staying asleep suffered from more severe LUTS. Our study found that a higher sleep quality score (which means worse sleep quality) was significantly associated with a high incidence of BPH, and men with the poorest sleep quality (Q4 group) were 1.615 times more likely to develop BPH than the Q1 group. Since the nocturia and lower urinary tract symptoms were most common symptoms to indicate benign prostatic hyperplasia, their studies supported our findings. While Araujo et al. defined poor sleep quality as sleep was restless, and Scovell focused on single sleep disorder symptoms, our study evaluated sleep disorders more comprehensively by five items, including the frequency of troubling falling asleep, getting back to sleep, waking too early, unrested after sleep and taking a nap during the day. In addition, Chou PS et al. [21] found that males with sleep apnea (SA) had 2.35-fold higher odds of BPH than men without SA, and Yang et al. [12] reported that reduced sleep duration increases the risk of BPH in Chinese people, which also emphasized the importance of sleep. Besides, several studies stated that nocturia symptoms of BPH might play a role in some sleep disorders, which indicated that the relationship between sleep problems and BPH might be bidirectional [19, 22, 23].

Although there was a strong correlation between sleep and BPH, the precise molecular pathways were unknown. Multiple rationales could apply. Sleep is typically regarded as a crucial component of the circadian rhythm. [24]Getting less sleep can lead to a.bnormalities of several core circadian clock genes PERIOD3 (PER3: a gene associated with diurnal preference) and PERIOD2 (PER2: a gene linked to morning preference). [25] Decreased PER 2 expression might hinder apoptosis and result in BPH, according to Li et al. [26] Furthermore, hormone metabolism in the body may be involved in this process. Androgens can directly affect prostate tissues and participate in BPH development. The presence of androgen, especially testosterone, was considered essential for BPH development. [27] Many studies have confirmed that androgens can directly affect prostate tissues and participate in BPH development. [28–30] Luboshitzky et al. demonstrated that the disruption of androgen secretion in older men may be caused by age-related sleep fragmentation. [31]Another study by Plamen found that the variability in the morning testosterone levels of healthy older men is significantly correlated with objectively observed variances in nightly sleep. [32] The length and duration of sleep significantly impact the fluctuation of the body’s hormone levels, particularly those of testosterone, making BPH that is reliant on androgen levels more likely to occur. Other factors, including autonomic nervous activity, and inflammatory cytokines may also play a role in the development of BPH through sleep disorders. [6, 33, 34]

Despite using a nationwide study, this study nevertheless had certain drawbacks. First, in contrast to more objective procedures such as prostatic ultrasonography, diagnosing BPH primarily depended on self-report, which might influence diagnosis. Second, the causal relationship between sleep quality and BPH may not be genuinely recognized due to the cross-sectional design of this study, and subsequent prospective and intervention studies may offer a more comprehensive explanation. Finally, recollection bias could exist for some covariates.

## Conclusion

Worse sleep quality was significantly associated with a higher incidence of benign prostatic hyperplasia among middle-aged and older Indian men. As the population ages at an unprecedented rate, it is advised that adults with sleep disorders pay attention to their prostatic condition early and improve their quality of life. Our study also offered evidence to prevent BPH risk by improving men’s sleep quality. A further prospective study is needed to clarify this association and explore potential mechanisms.

## Electronic supplementary material

Below is the link to the electronic supplementary material.

Supplementary Material 1

## Data Availability

Publicly available datasets were analyzed in this study, which can be found at: https://www.iipsindia.ac.in/content/data-request. And the sorted data was provided as **Additional file 1**.

## References

[1] Berry SJ, Coffey DS, Walsh PC, Ewing LL (1984). The development of human benign prostatic hyperplasia with age. J Urol.

[2] Verhamme KM, Dieleman JP, Bleumink GS, van der Lei J, Sturkenboom MC, Artibani W, Begaud B, Berges R, Borkowski A, Chappel CR (2002). Incidence and prevalence of lower urinary tract symptoms suggestive of benign prostatic hyperplasia in primary care–the Triumph project. Eur Urol.

[3] Barry MJ, Fowler FJ, O’Leary MP, Bruskewitz RC, Holtgrewe HL, Mebust WK, Cockett AT (1992). The american Urological Association symptom index for benign prostatic hyperplasia. The Measurement Committee of the american Urological Association. J Urol.

[4] Chughtai B, Forde JC, Thomas DD, Laor L, Hossack T, Woo HH, Te AE, Kaplan SA (2016). Benign prostatic hyperplasia. Nat reviews Disease primers.

[5] Tufik S, Andersen ML, Bittencourt LR, Mello MT (2009). Paradoxical sleep deprivation: neurochemical, hormonal and behavioral alterations. Evidence from 30 years of research. Anais da Academia Brasileira de Ciencias.

[6] Sönnerqvist C, Brus O, Olivecrona M (2021). Validation of the scandinavian guidelines for initial management of minor and moderate head trauma in children. Eur J trauma Emerg surgery: official publication Eur Trauma Soc.

[7] Everson CA, Crowley WR (2004). Reductions in circulating anabolic hormones induced by sustained sleep deprivation in rats. Am J Physiol Endocrinol metabolism.

[8] Hipólide DC, Suchecki D, Pimentel de Carvalho Pinto A, Chiconelli Faria E, Tufik S, Luz J (2006). Paradoxical sleep deprivation and sleep recovery: effects on the hypothalamic-pituitary-adrenal axis activity, energy balance and body composition of rats. J Neuroendocrinol.

[9] Gooren L (2003). Androgen deficiency in the aging male: benefits and risks of androgen supplementation. J Steroid Biochem Mol Biol.

[10] Weiss JP, Blaivas JG, Stember DS, Brooks MM (1998). Nocturia in adults: etiology and classification. Neurourol Urodyn.

[11] Li Y, Zhou X, Qiu S, Cai B, Wang S, Chen L, Hu D, Jiang Z, Wang M, Xiong X (2022). Association of sleep quality with lower urinary tract symptoms/benign prostatic hyperplasia among men in China: a cross-sectional study. Front Aging Neurosci.

[12] Xiong Y, Zhang Y, Zhang F, Wu C, Qin F, Yuan J (2022). Reduced sleep duration increases the risk of lower urinary tract symptoms suggestive of benign prostatic hyperplasia in middle-aged and elderly males: a national cross-sectional study. The aging male: the official journal of the International Society for the Study of the Aging Male.

[13] Koskderelioglu A, Gedizlioglu M, Ceylan Y, Gunlusoy B, Kahyaoglu N (2017). Quality of sleep in patients receiving androgen deprivation therapy for prostate cancer. Neurol sciences: official J Italian Neurol Soc Italian Soc Clin Neurophysiol.

[14] Speakman M, Kirby R, Doyle S, Ioannou C (2015). Burden of male lower urinary tract symptoms (LUTS) suggestive of benign prostatic hyperplasia (BPH) - focus on the UK. BJU Int.

[15] Bloom DE, Sekher TV, Lee J (2021). Longitudinal aging study in India (LASI): new data resources for addressing aging in India. Nat Aging.

[16] Jenkins CD, Stanton B-A, Niemcryk SJ, Rose RM (1988). A scale for the estimation of sleep problems in clinical research. J Clin Epidemiol.

[17] Banerjee S, Boro B (2022). Analysing the role of sleep quality, functional limitation and depressive symptoms in determining life satisfaction among the older Population in India: a moderated mediation approach. BMC Public Health.

[18] Cohen A, Dias A, Azariah F, Krishna RN, Sequeira M, Abraham S, Cuijpers P, Morse JQ, Reynolds CF 3rd, Patel V. Aging and well-being in Goa, India: a qualitative study. Aging Ment Health. 2018;22(2):168–74. 10.1080/13607863.2016.1236239 PMC5374050 27689842

[19] Araujo AB, Yaggi HK, Yang M, McVary KT, Fang SC, Bliwise DL (2014). Sleep related problems and urological symptoms: testing the hypothesis of bidirectionality in a longitudinal, population based study. J Urol.

[20] Scovell JM, Pastuszak AW, Slawin J, Badal J, Link RE, Lipshultz LI (2017). Impaired sleep quality is Associated with more significant lower urinary tract symptoms in male Shift Workers. Urology.

[21] Chou PS, Chang WC, Chou WP, Liu ME, Lai CL, Liu CK, Ku YC, Tsai SJ, Chou YH, Chang WP (2014). Increased risk of benign prostate hyperplasia in sleep apnea patients: a nationwide population-based study. PLoS ONE.

[22] Oelke M, Fangmeyer B, Zinke J, Witt JH (2018). [Nocturia in men with benign prostatic hyperplasia]. Aktuelle Urologie.

[23] Bal K, Ayik S, Issi Y, Bolukbasi A, Akhan G (2012). Sleep analysis of patients with nocturia and benign prostatic obstruction. Urology.

[24] Jagannath A, Taylor L, Wakaf Z, Vasudevan SR, Foster RG (2017). The genetics of circadian rhythms, sleep and health. Hum Mol Genet.

[25] Goel N, Basner M, Rao H, Dinges DF (2013). Circadian rhythms, sleep deprivation, and human performance. Prog Mol Biol Transl Sci.

[26] Li Y, Shi B, Dong F, Zhu X, Liu B, Liu Y (2019). Effects of inflammatory responses, apoptosis, and STAT3/NF-κB- and Nrf2-mediated oxidative stress on benign prostatic hyperplasia induced by a high-fat diet. Aging.

[27] Devlin CM, Simms MS, Maitland NJ (2021). Benign prostatic hyperplasia - what do we know?. BJU Int.

[28] Sasagawa I, Nakada T, Kazama T, Satomi S, Terada T, Katayama T (1990). Volume change of the prostate and seminal vesicles in male hypogonadism after androgen replacement therapy. Int Urol Nephrol.

[29] Salter CA, Mulhall JP (2019). Guideline of guidelines: testosterone therapy for testosterone deficiency. BJU Int.

[30] Pejčić T, Tosti T, Tešić Ž, Milković B, Dragičević D, Kozomara M, Čekerevac M, Džamić Z (2017). Testosterone and dihydrotestosterone levels in the transition zone correlate with prostate volume. Prostate.

[31] Luboshitzky R, Zabari Z, Shen-Orr Z, Herer P, Lavie P (2001). Disruption of the nocturnal testosterone rhythm by sleep fragmentation in normal men. J Clin Endocrinol Metab.

[32] Penev PD (2007). Association between sleep and morning testosterone levels in older men. Sleep.

[33] Navaneethakannan M, Nandeesha H, Sreenivasan SK (2022). Relationship of interleukin-23 with matrix metalloproteinase-9, pentraxin-3, sleep quality and prostate size in benign prostatic hyperplasia. Andrologia.

[34] Roehrborn CG (2008). Pathology of benign prostatic hyperplasia. Int J Impot Res.

